# Eosinophilic disorders of the gastro-intestinal tract: an update

**DOI:** 10.1186/s12948-016-0055-y

**Published:** 2016-12-01

**Authors:** Erminia Ridolo, Valerie Melli, Gianluigi De’ Angelis, Irene Martignago

**Affiliations:** Department of Clinical and Experimental Medicine, University of Parma, via Gramsci, 14, 43100 Parma, Italy

**Keywords:** Eosinophilic esophagitis, Eosinophilic gastroenteritis, Pathogenesis, Diagnosis, Therapy

## Abstract

Eosinophilic diseases of the gastrointestinal tract, including eosinophilic esophagitis (EoE) and eosinophilic gastroenteritis (EGE), are rare chronic pathologies of the digestive system, with an immuno-mediated pathogenesis. Recent data suggest that, together with the “classic” IgE-response to allergens, also a delayed hypersensitivity mechanism could be involved in the development of eosinophilic disorders. EoE and EGE were studied only in the latest decades and as a consequence accurate data are not yet available, concerning not only pathogenesis, but also epidemiology, treatment and outcomes. The diagnosis of EoE is centered on endoscopic findings but the certainty is obtained by histological examination from biopsy samples, that has a sensitivity of 100% when based on five samples. The currently available treatments include topical corticosteroids, specific diets and endoscopic treatment. Concerning EGE, three subtypes (mucosal, muscular, and serosal) were identified. The diagnosis is based, as for EoE, on endoscopic and histological assessment, and the treatment includes pharmacological and dietetic approaches. Further studies are warranted in order to better define the etiology and pathogenesis of eosinophilic diseases of the gastrointestinal tract, and thus to develop more appropriate and specific therapies.

## Background

Primitive eosinophilic esophagitis (EoE) and eosinophilic gastroenteritis are part of a group of diseases first described in 1937 by Kaijser and called EGIDs (eosinophilic gastrointestinal diseases) [1]. These clinical entities are characterized by selective infiltration of gastrointestinal tract by eosinophils, in absence of other causes of known eosinophilia.

Interest in EGIDs dates back to the latest decades and, also due to the rarity of these disorders, the data about epidemiology, pathogenesis, therapy and outcomes thus far available are partial. There is an urgent need to better characterize EGIDs, to understand the etiology and to define a more precise therapeutic approach. In fact, current treatments are yet controversial and there is no consensus on how long the diet and/or steroid therapy should be maintained.

## Eosinophilic esophagitis

EoE is a chronic inflammatory disease of the esophagus with an immuno-allergic pathogenesis, characterized by eosinophilic infiltration (≥15 Eo/high power field [HPF]) and secondary fibrosis [1]. EoE was once considered a rare clinical entity and in fact it was relatively unknown until the ‘90s, while now it is of increasing interest.

In United States and Europe epidemiological studies found a prevalence of EoE of 50 patients per 100.000 inhabitants [2].

EoE can affect any age, from infancy to old age, but diagnosis is more frequent in third/fourth decades [3]. Male subjects are more frequently affected than females, with a rate of 3:1 [2, 3]. The reason for this gender difference is not yet explained, but a single nucleotide polymorphism (SNP) in the gene for thymic stromal lymphopoietin (TSLP), was identified on chromosome Xp22.3 and also on chromosome Yp11.3. These findings can be a possible explanation for the prevalence of EoE in males [4]. Caucasian ethnicity is more commonly interested in EoE, with a reported predominance of 90% in some case studies. However, the disease can affect also African American, Asian, Native American and Hispanic populations [5].

## Pathogenesis

EoE pathogenesis is complex with many factors involved, particularly genetic and environmental, and the precise mechanism is still unknown. Environmental factors seems to play a significant role in development of EoE: in fact, though the heritability rate is about 70%, concordance between monozygotic twins is only 30%, suggesting a powerful role for environmental factors (81.0%) [6]. An important epidemiological study, conducted in Cincinnati (US) evaluated 6108 individuals from 1366 families of patients with EoE: the recurrence risk ratio was found to be increased 10- to 64-fold compared with the general population, with an incidence of EoE in relatives from 1.8 to 2.4%, depending on relationship and sex [6].

In literature, an association between EoE and some hereditary collagen alterations, such as Marfan and Enhlers-Danlos syndromes, was reported [7]. More recently another associated disease was reported: three patients with EoE suffered from hypertrophic cardiomyopathy, leading the Authors to suggest a possible genetic alteration underling both diseases [8].

Several genetic alterations have been identified in patients with EoE. The most common involved gene is the one for eotaxin-3, a chemokine active on eosinophils, that plays a fundamental role in EoE. In fact, it is expressed in esophageal mucosa 53 times more than in healthy subjects. The alteration is represented by SNP in the 3′ untranslated region of eotaxin-3 gene [5, 9].

Another important mutation concerns the gene for TSLP, and its risk alleles, such as rs3806932, present on chromosome 5q22. TLSP is a cytokine produced by epithelial cells, whose action consists of inducing a Th2 cell-mediated response in dendritic cells [10–12]. Also a SNP for TLSP-receptor seems to be involved and its presence on chromosome Y could be a possible cause of male prevalence for EoE [11].

A recent study suggested a possible mechanism underling the role of TLSP for development of EoE. According to these results specific food antigens (hen’s egg ovalbumin) can trigger TSLP secretion by differentiated epithelial cells in esophagus [13].

While eotaxin-3 and TLSP are responsible for epithelial dysregulation, also the barrier function is altered in EoE. In this case other two proteins are involved: filaggrin and desmoglein-1.

A SNP of the gene for filaggrin (2282del4) was analyzed in a cohort of 365 patients with EoE compared to 165 healthy subjects, showing a significantly association with the disease (OR = 5.0) [11]. The hyperplasia of esophageal epithelial cells observed in patients with EoE is secondary to decreased expression of genes that regulate epidermal differentiation. One of these lacking proteins is desmoglein 1 (DSG1), whose action is important to maintain an effective barrier in the esophagus [14]. Moreover, downregulation of DSG1 promotes upregulation of periostin, an extracellular matrix protein that is involved in fibrosis of the esophagus tissue [15].

The eosinophils’ role is unquestionable, due to the production of potent pro-inflammatory mediators, such as cytokines, GM-CSF, TGF-β and TNF-α, Th2 lymphocytes and their cytokines (IL-5, IL-4, IL-13) that are main actors in coordinating adaptive immunity in the pathogenesis of EoE. IL-5 is the most important mediator for eosinophilic activation and recruitment. Its action is confirmed by a study demonstrating its overexpression in esophageal biopsies from pediatric patients with EoE [16]. IL-13 is produced primarily by Th2 cells and its activation leads to an increased generation of eotaxin-3. High concentrations of IL-13 were detected in esophageal biopsies [17]. IL-4 stimulates, together with IL-13, the production of eotaxin-3 [12].

Recently a genome-wide genetic association of EoE was identified at 2p23 spanning, the calpain 14 (CAPN14) gene. CAPN14 is a gene induced by IL-13 that encodes for calpain 14, a cysteine-protease, which is overexpressed in esophageal biopsies of patient with EoE [12, 18]. The role of calpain 14 is not clearly defined, but its overexpression results also in loss of desmoglein 1 [18].

IL-13 is responsible to increase the expression of other genes involved in EoE pathogenesis, such as BANCR (BRAF-activated non-coding RNA) and NTRK1 (Neurotropic Tyrosine Kinase Receptor type 1). RNA sequencing in esophageal biopsies identified an overexpression of lcnRNA BANCR, that is responsible for local hypereosinophilia. BANCR is also related to the increase of periostin production [19]. NTRK1 is an early transcription target of IL-13 and its expression is higher in esophageal tissue of patients with EoE than in healthy subjects. Its increased concentration determines an augmented response to its ligand NGF (Neurotropic Tyrosine Kinase) that has a primary role in hyperplasia [20]. IL-13 is also responsible of inducing production of chemokines CCL11, CCL24 AND CCL26 from eotaxins [5]. Other genetic variants in the cluster of IL-5 and IL-13 regions have been identified, but their role is not clearly defined [12]. New actors of EoE pathogenesis were recently identified: mast cells and invariant natural killer T (iNKT).

Mast cells seem to contribute to EoE releasing prostaglandin D2 that is able to recruit eosinophils in esophagus [21]. In fact, in animal models of EoE, mast cells are increased in number after allergen stimulation, proportionally with time and dose of the stimulus [22].

iNKT are a subpopulation of T cells, whose concentration in esophageal biopsies of patients with EoE is increased compared to healthy subjects. These cells are able to recognize the glycolipid antigen and this may explain their role in development of EoE: iNKT cell-deficient CD1d-null mice were protected from food allergen-induced EoE, while in the same animals iNKT activation is sufficient to induce EoE. In fact, their activation induces the release of inflammatory cytokines, including IL-4, IL-5 and IL-13 [23]. Moreover, a recent study suggests that sphingolipids of milk may stimulate iNKT cells to produce these cytokines [24].

Another mediator released by eosinophils and implicated in EoE pathogenesis is TGF-β1. It mediates the epithelial cell proliferation and extracellular remodeling, through the upregulation of genes involved in fibrosis, i.e. matrix metalloproteinase 2 [12]. Also leukotriene C4 may contribute to worsen fibrosis through its metabolites LTD4 and LTE, that are able to stimulate smooth cells contraction [5].

The pathogenetic role of atopy was confirmed by experimental and epidemiological studies. EoE is associated with a condition of atopy in 75% of patients [5]. Sensitization regards inhalant and/or food allergens (in adults 86–93% towards inhalants and 50–82% towards food allergens). Sensitization is associated with allergic clinical presentations in 68% of adult patients: the most frequent symptoms are respiratory (allergic rhinitis 60% approximately, allergic asthma 39%) [25, 26]. In pediatric patients a family history of atopy was present in 73.5% [26]. The important effect of food allergy in patients affected by EoE is demonstrated indirectly by the improvement of symptoms after elimination of allergenic food from diet, as we will discuss below. In addition, development of EoE was described as a complication of oral tolerance induction for food allergens. Ridolo et al. reported how EoE occurred in a 10 year-old child after acquiring tolerance for egg with an oral protocol of induction. The patient presented dyspepsia and dysphagia 5 months after egg reintroduction in diet, with an endoscopic and histological demonstration of EoE. Symptoms disappeared persistently after elimination of egg from the diet [27]. Other cases were described after administration of oral specific immunotherapy for milk, egg or peanuts [28, 29]. Notably, only a small part of EoE patients with food IgE- sensitization presents with anaphylaxis. The protection mechanism underlying this aspect is not yet well defined, but a recent study highlighted the conceivable role of IgG4 in the development of EoE. In fact, an increase in production of IgG4 can be detected in lamina propria of esophagus in biopsies of patients with EoE [30]. This hypothesis was formulated after finding that omalizumab did not reduce nor symptoms of EoE neither tissue eosinophilia compared to placebo in a prospective trial. Moreover the Authors observed granular deposits of IgG4, plasma cells containing IgG4 and serum levels of IgG4 reactive to specific foods and suggested that EoE could be an IgG4-associated pathology [30]. Further studies are warranted to confirm these findings.

Regarding the role of inhalant allergens in development of EoE, the data in favor of their involvement are certain. In a study of Yamazaki et al. mononuclear cells from 25 patients with EoE were incubated with inhalant allergens (dust mites, *Aspergillus fumigatus*, *Alternaria alternata*, and ragweed pollen): significantly higher levels of IL-5 and IL-13 were detected in response to the allergens, compared than healthy controls, even in the absence of an evident IgE-mediated sensitization [31]. Another study on animal models demonstrated that intranasal exposure to inhalant allergen (*A. fumigatus*) in sensitized mice induced esophagus eosinophilia, eosinophilic degranulation and epithelial hyperplasia [32].

Furthermore, a study performed in patients with seasonal respiratory allergy established a correlation between esophageal eosinophilic infiltration and seasonal symptoms [33]. In fact a seasonal distribution of new diagnoses of EoE was demonstrated: a 1-year study highlighted that in 41 adults patients, a new EoE diagnosis was more frequent in spring months (44%, 18 of 41 patients) [10], while a 6-years-long retrospective analysis of a pediatric population confirmed that new diagnosis were significantly less frequent in winter than in other seasons [11]. A retrospective Australian study reported a higher recurrence of esophageal food bolus during the pollen season in patients with EoE [12].

Moreover patients with EoE presents an high prevalence of sensitizations to pan-allergens, responsible for cross-reactivity among inhalant and food allergens. In particularly, sensitization to PR-10, the birch allergen Bet v 1, seems to be very frequent: in a study of Van Rhijn 39% of patients presents sensitization to this molecule [34]. In a recent study the association between bolus as first symptom of EoE, more severe eosinophilic infiltration and sensitization to PR-10 has been established [35].

Of note, there are differences in the sensitization patterns among children and adult population affected by EoE, with a progressive increase in inhalant allergy with age and a parallel diminution in food sensitization. These differences, although not specific for EoE, are correlated with patient age and have important therapeutic implications, suggesting a predominant role of food allergens in children.

## Clinical presentation

As mentioned above, EoE can affect any age, but clinical presentation differs between children and adult patients [26]. In adults the most common symptoms are dysphagia, food bolus, thoracic and epigastric pain, the onset being similar to a PPI (Pomp Proton Inhibitors)—not responding GERD [26]. In pediatric patients the most common manifestations are food refusal and growth retardation in infants, and vomit, nausea and abdominal pain in older children [4]. It is important to ascertain also the presence of coping mechanism that patients can apply during swallow in order to overcome symptoms [36].

## Endoscopic findings

**Fig. 1:**
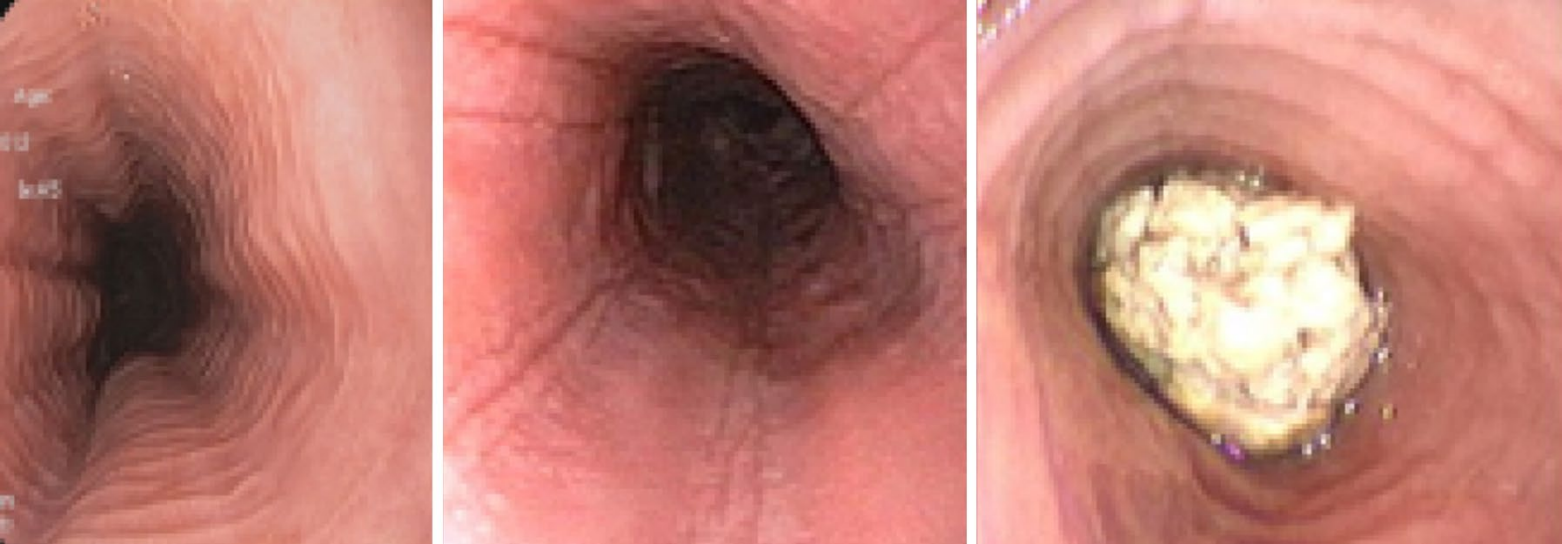
EoE

The possible macroscopic findings during gastroscopy are: mucosa edema, white exudates, longitudinal grooves, concentric rings (“trachealization” of the esophagus), and stenosis. In about 20% of cases esophagus may, however, appear macroscopically normal. A meta-analysis including 4678 patients with EoE and 2742 controls, estimated the following frequency of the endoscopic features: trachealization in 44%, linear grooves in 48%, white exudates in 27%, stenosis in 21% and reduction in caliber 9% [37]. None of these endoscopic features is pathognomonic of EoE: sensitivity is low, varying between 15% for stenosis and 48% for esophagus rings, while the specificity ranged between 90% for pale mucosa and 95% for stenosis [37] (Fig. 1).

## Histology

Esophagus is the only gastrointestinal organ that does not present eosinophils in normal condition. The number of eosinophil present in mucosa increases progressively from stomach to cecum [38]. Even if finding eosinophils in esophagus is a pathologic sign, it is not pathognomonic of EoE. In fact, other clinical entities, such as gastroesophageal reflux disease (GERD), eosinophilic gastroenteritis, parasitosis, celiac disease, Crohn disease, drugs hypersensitivity, vasculitis, idiopathic hypereosinophilic syndrome, Graft vs Host disease, and scleroderma, can present eosinophilic infiltration in esophagus [39].

**Fig. 2:**
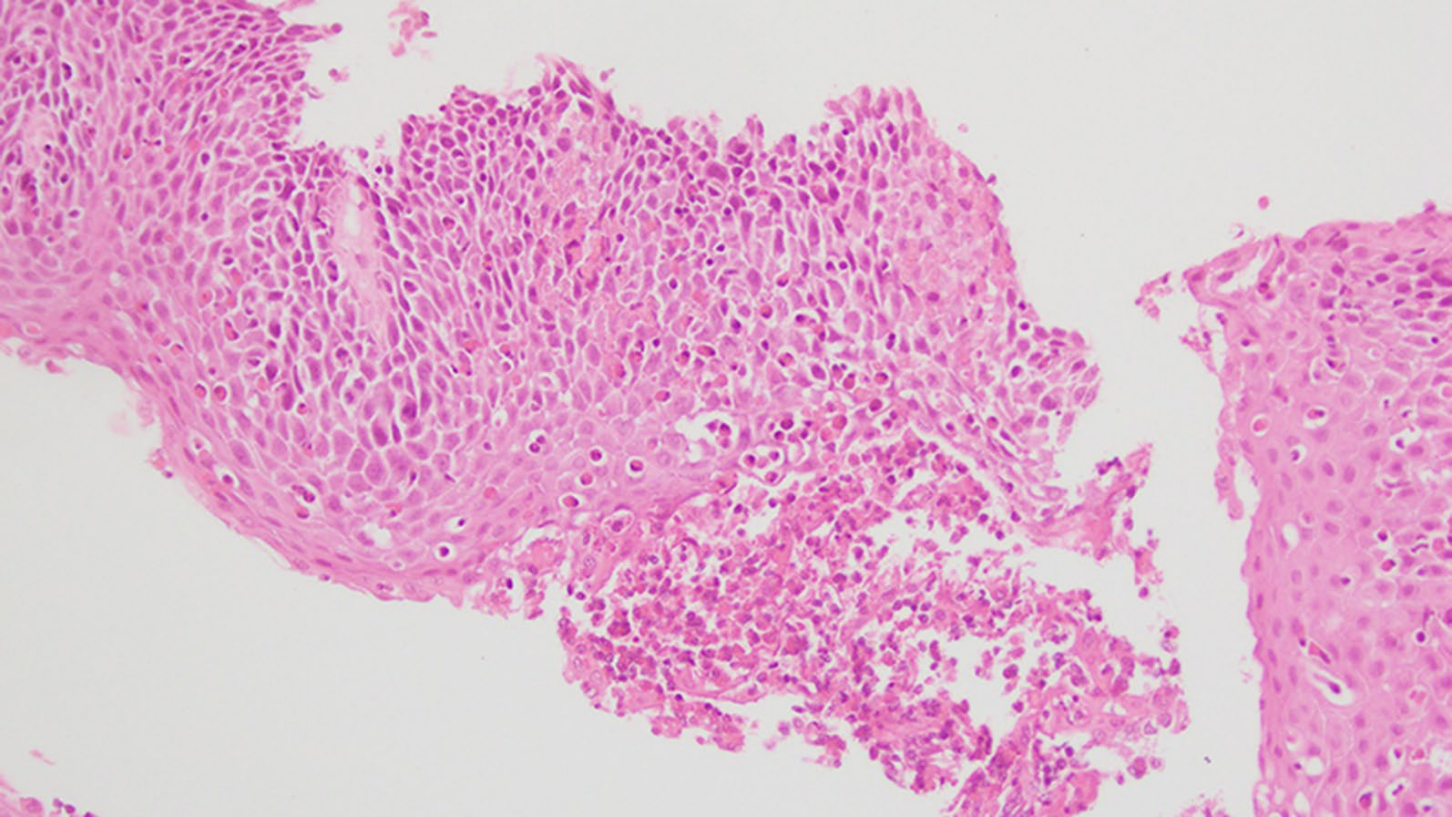
Hematoxylin-eosin, 20× magnification: Frustule of esophageal mucosa with diffuse infiltration of eosinophils (Eo > 20/HPF), microabscesses, disruption of the epithelial barrier

**Fig. 3:**
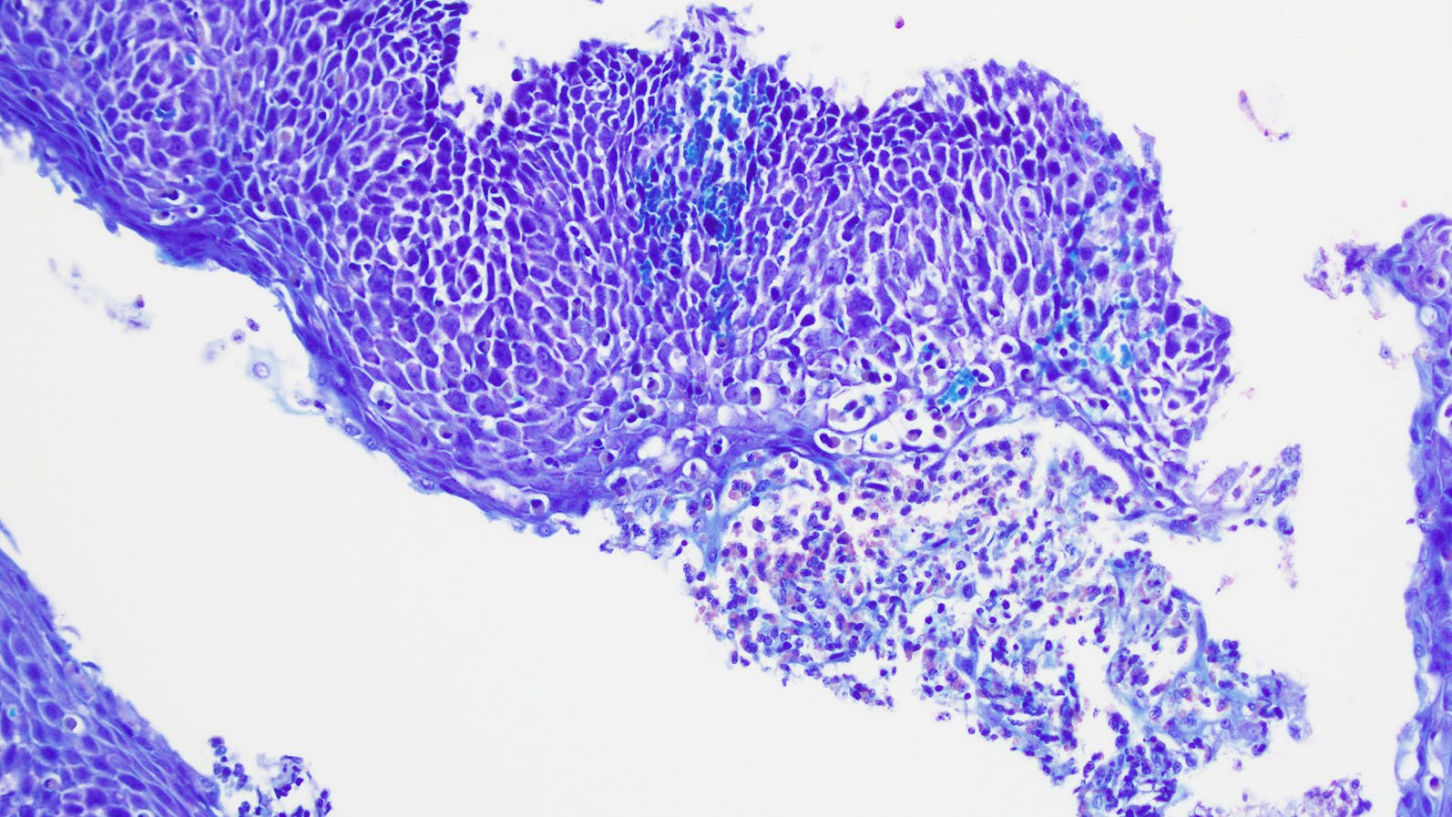
Same sample of Fig. 2 in Giemsa, 20× magnification: diffuse eosinophilic infiltration with microabscesses, it can be appreciate also the intercellular edema and the acantholysis resulting from the eosinophils’ degranulation

First of all, it is necessary to perform histology only after at least 8 weeks of therapy with PPI to make diagnosis of EoE. Then biopsy must present ≥15 eosinophils/HPF in at least one sample. Other characteristic findings are microabscesses, elongation of the papillae, hyperplasia of the basal layer, detachment of the surface layers, degranulation of eosinophils, increased number of mast cells and lymphocytes [40] (Figs. 2, 3).

## Diagnosis

The symptoms mentioned above are not pathognomonic of EoE. Also sensibility, specificity and predictive value of the endoscopic features are insufficient to make a certain diagnosis [41]. Biopsy of esophagus is always necessary, even when the macroscopic appearance is normal. A study conducted on 66 adults patients confirmed that sensibility of histological examination was 100% when based on the analysis of five samples, while it was only 55% if even a single sample was withdrawn [42]. These data were validated also in pediatric population, in which sensibility was 73, 84, 95 and 100% for 1, 2, 3 and 6 biopsies, respectively [43]. Thanks to studies like these, also guidelines recommend to perform multiple biopsies in proximal, medium and distal esophagus [5]. It is also strongly suggested to perform biopsies also in stomach and duodenum in order to exclude eosinophilic gastroenteritis. At least 15 or more eosinophils/HPF must be found in case of EoE. As mentioned above, eosinophils are not present in esophagus in normal condition, but this finding is possible also in other pathologies. In particular two pathologies can present isolated eosinophilic esophagitis: GERD and PPI-responsive eosinophilic esophagitis. In order to distinguish them from EoE, a further biopsy after an 8-week-long therapy with PPI must be done. In case of absence of clinical and histological response, it is possible to set a diagnosis of EoE [40].

It is unclear whether the esophageal eosinophilia responsive to PPI is a variant of gastroesophageal reflux or a variant of EoE. A small series of four pediatric patients (mean age 9.5 years) with esophageal eosinophilia (mean 52 eosinophils/HPF), initially responsive to PPI, showed an evolution towards primitive EoE [44]. The reason of the initial response to PPI is unclear, though a possible anti-inflammatory activity, and for omeprazole also the capacity to block in vitro the secretion of eotaxin-3 from esophageal cells, should be involved [45].

## Therapy

Treatment of EoE includes pharmacologic, dietetic and endoscopic therapy. The appropriate therapy must be evaluated according to symptoms and time of response with a step-up/step down process. Dietetic and pharmacologic treatments are often required for long periods as EoE is a chronic disease. No fixed timing is standardized and the duration must be customized to each single patient and his/her response.

### Pharmacologic therapy

An 8-weeks-long therapy with PPIs is necessary to rule out GERD or eosinophilic esophagitis responsive to PPI, before an EoE diagnosis can be made.

First line therapy for EoE is represented by topical glucosteroids. A great part of patients presents both clinical and histological improvement with this treatment [46]. Topical treatment has an anti-inflammatory action thanks to ability in inducing some anti-inflammatory genes, such as FK-506 binding protein and miRs, and in reversing the eosinophilic transcriptome [5].

The molecules more frequently prescribed for EoE are budesonide and fluticasone. Industry does not provide specific formulation for EoE and then available devices for asthma must be prescribed to patients. Therefore, clinicians must educate subjects to swallow and not to inhale corticosteroids in order to have a topic action on esophagus.

Doses of fluticasone usually vary between 88 and 440 mcg/die in children and between 880 and 1760 mcg/die in adults, while budesonide is 1 mg in children and 2 mg/die in adults [1]. Only 1% of these swallowed steroids is absorbed and consequently systemic side effects are very rare. The most common unwanted effect is oral and esophageal candidosis, that can be prevented educating patients to rinse their mouth after therapy [47].

Occasionally, discrepancy between histological response and clinical improvement can be found. In fact, some patients continue to present symptoms even if endoscopic test is negative. This is probably secondary to the pre-existing fibrosis and muscle hypertrophy [5]. Pediatric patients are indeed more responsive to topical steroids because adults have already a significant remodeling of esophagus before diagnosis and treatment are performed [48].

Systemic steroids are also very effective to improve symptoms, but their use must be limited to the most severe cases (i.e. important weight loss secondary to dysphagia and/or esophagus stenosis). The recommended dose is 1–2 mg/kg (max 60 mg/24 h) for 7–15 days [44]. Recurrence of EoE is more frequent after withdrawal of systemic steroids [47].

Among the other drugs that were evaluated there are biological drugs such as monoclonal antibodies directed towards IL-5, IL-13, CRTH2 and IgE, immunomodulators and leukotriene inhibitors. Immunomodulators, such as azathioprine and 6-mercaptopurine were used in three patients with corticosteroid-dependence obtaining long-term remission of EoE, but their use is not currently recommended [49]. In contrast, montelukast improves clinical symptoms, but is not able to gain histological improvement [50].

Omalizumab, an antibody against IgE, failed in gaining both symptoms control and eosinophilic infiltration in esophageal biopsies [30]. Antibodies against IL-5 (mepolizumab and reslizumab) were selected to treat EoE patients due the fundamental role of this cytokine in development and activation of eosinophils, but only a limited number of patients have an acceptable response to them. Mepolizumab was administered to 11 adults [51] and to 45 children [52] in two different trials, obtaining decrease of eosinophilic infiltration, but not a significant improvement in clinical manifestations. A double-blind placebo controlled study on the use of reslizumab was performed on 226 children and adolescents, but the difference in improvements between treated and placebo groups was not statistically significant [53]. A recent trial studied the effectiveness of QAX576, anti-IL-13 antibody, in 23 adult patients, demonstrating a significantly decrease of intraepithelial esophageal eosinophilia and of the expression of EoE-relevant esophageal transcripts, such as eotaxin-3, inducing a sustained clinical improvement [12, 54].

### Dietetic therapy

Dietetic therapy of EoE can be effective and it is usually prescribed initially together with pharmacologic treatment, even though it is often recommended after drugs discontinuation.

Three different treatments have been used during the last decades:

1. Elemental diet, during which patients are fed only with elemental amino-acid based formula;
2. IgE targeted diet, during which patients must eliminate only food to which they are sensitized.
3. SFED (Six Food Elimination Diet), in which patients must exclude the six more probable food allergens: milk and dairy products, egg, soy, tree nut/peanut, sea food (fish/shellfish);

The first type of diet seems to be very effective to induce clinical an histological remission, if given for at least 6 weeks [47]. In 1995 Kelly et al. was the first to demonstrate that a diet based almost exclusively on amino acids (only exceptions were corn, apple and clear liquids) was successful in obtaining a complete resolution of clinical manifestations in 8 of 10 children with EoE. The remaining two still achieved a significant clinical improvement, and children presented a complete histological remission [55]. Peterson et al. confirmed these data in an adult population of 18 patients: 13 obtained a full histological response and 4 presented a reduction of 50% of the eosinophilia in esophagus [56].

A recent meta-analysis confirmed the efficiency of the elemental diet in achieving histologic remission in both children and adults in about 90% of the cases [57]. The most important limitation of this diet is its strong impact on the quality of life. In fact, patients are required to drink great amount of liquid, unpalatable and very expensive formulas [5, 47].

The second dietetic approach is based on elimination of foods to which the patient is sensitized. A great variability in remission rates has been highlighted in a meta-analysis, so that the Authors stated that these data are likely to support the involvement of a cell-mediated delayed hypersensitivity reaction, instead of an IgE-mediated mechanism underlying EoE pathogenesis [57].

In another study, the foods that clinically caused symptoms to patients were compared to sensitizations identified towards food with skin prick test (SPT). In these patients SPT were able to identify the actual trigger food only in 13% of cases [58]. Anyway, a target diet with elimination of foods inducing positive results to SPT and atopy patch test was effective in 26 children with EoE. The foods most frequently positive to SPTs are milk, egg, peanuts, seafood, peas, rye, tomato and wheat, while the most common positivity to atopy patch tests were wheat, corn, beef, milk, soy, rye, chicken, oats and potato [58]. In adults, the effectiveness of this type of diet is not yet clearly demonstrated.

The third dietetic approach is SFED diet. In 2006 Kagalwalla proposed the empiric use of SFED, starting from foods most frequently involved in children allergy [59]. The efficacy of SFED was about 70% according to a meta-analysis. Such analysis documented that the most commonly causative foods involved in relapsing of symptoms after their reintroduction were cow’s milk, wheat, eggs, and soy/legumes [57].

### Endoscopic treatment

In case of severe stenosis (diameter < 10 mm), in patients not responsive to pharmacological and/or dietetic therapy, endoscopic dilatation leads to a rapid improvements of clinical manifestations. In some cases this remission can last several months, but multiple sessions are needed to achieve an optimal dilatation. At least an esophageal diameter of 15–18 mm must be reached to obtain symptoms relief. The risk of perforation after dilatation is low, about 0, 1%. Obviously this treatment does not have any effect on pathogenetic and inflammatory process [47].

## Natural history

Knowledge on long-term prognosis of EoE is actually limited. Available data unfortunately indicate a progressive trend towards esophageal fibrosis/stenosis.

A retrospective study in 200 patients highlighted that diagnosis’ delay is associated with a higher prevalence of fibrostenotic form (from 46.5% for diagnostic delay between 0 and 2 years to 87.5% for diagnostic delay >20 years) and is the only factor associated with an increased risk of stenosis [60]. These results were also confirmed by another recent retrospective analysis: Dellon et al. indicated that for every age increase of 10 years the risk of having a fibrostenotic form doubled [61]. Spergel et al. followed 620 pediatric patients for over 14 years, showing that EoE persisted in the vast majority of cases. Fortunately there was not even a case of progression to any other gastrointestinal disease [62]. In literature there are no reported cases of progression to neoplastic diseases. Actually we can state that, when the diagnosis is made during childhood, EoE seems to persist throughout adult life, but follow-up studies in the long term are not yet available.

## Eosinophilic gastroenteritis

Eosinophilic Gastroenteritis (EGE) is a rare and idiopathic group of diseases characterized by eosinophilia in one or more tracts of gastrointestinal system. If eosinophilia is limited to stomach, it is defined as eosinophilic gastritis; if it is limited to colon, it is called eosinophilic colitis. Clinical manifestations are different and never specific of this spectrum of disease, also according to the interested tract [46].

The precise incidence is unknown, due to EGE rarity, but it is rising in the latest years, probably because of a better recognition and an increasing interest [63]. Nowadays, incidence is estimated about 1–30/100.000, but it is probably underestimated [46].

Adults seem to be more affected by EGE then children, with a maximum incidence in the fourth–fifth decades. Male are slightly more interested than woman (3:2), with the exception of serosal subtype. Even in this case, EGE is more common in Caucasians [46, 64].

About 70% of patients suffering from EGE present a personal or familiar history of atopy. The most common allergies presented by these patients are food sensitizations [65].

## Pathogenesis

EGE etiology and pathogenesis are not clearly defined. Delayed type hypersensitivity to food is the most probable underlying mechanism, according to the current data. In these patients hypereosinophilia found in peripheral blood and in gastrointestinal tissue, increased serum levels of IgE, presence of elevated mediators produced by eosinophils in intestinal biopsies and response to steroids therapy are supporting this pathogenetic hypothesis [46].

## Classification and clinical manifestations

Anatomic localization of eosinophilic inflammation influences clinical manifestations, that can vary from nausea to intestinal obstruction. As a consequence, EGE is classified according to the most infiltrated layer of the intestinal wall in [66]:

1. *Mucosal* the most common subtype. Patients have non-specific symptoms, such as nausea, vomiting, diarrhea, anemia due to chronic inflammation or iron deficiency, clinical manifestations secondary to malabsorption. In particular, in children weight loss and protein losing enteropathy are the most common manifestations [40, 63].
2. *Muscular* the second form for frequency. The interest of this layer often leads to intestinal obstruction secondary to the thickening of intestinal wall [67]. The most common is jejunal obstruction [38]. Colo-colonic intussusceptions are rare [68].
3. *Serosal* the most uncommon subtype, even though is the most frequent in females [40]. Clinical presentation begins usually with ascites, sometimes associated with symptoms of intestinal obstruction. Ascites is characterized by marked eosinophilia in ascitic fluid. Serosal subtype differs also for its consistent response to corticosteroids therapy [40, 63].

EGE subtypes, correlate with prognosis. Serosal pattern in fact does not present a continuous chronic course, but mostly single episodic flares up, with long disease-free periods. Instead, mucosal subtype is characterized by a persistent presence of symptoms and muscular pattern presents frequent relapses [69].

It must be noted anyway that it is difficult in everyday practice to distinguish which is the most involved layer for two reasons: overlap among 2 or 3 subtypes is very common and often only mucosal and submucosal biopsies are taken [69].

## Endoscopic and histological aspects

**Fig. 4:**
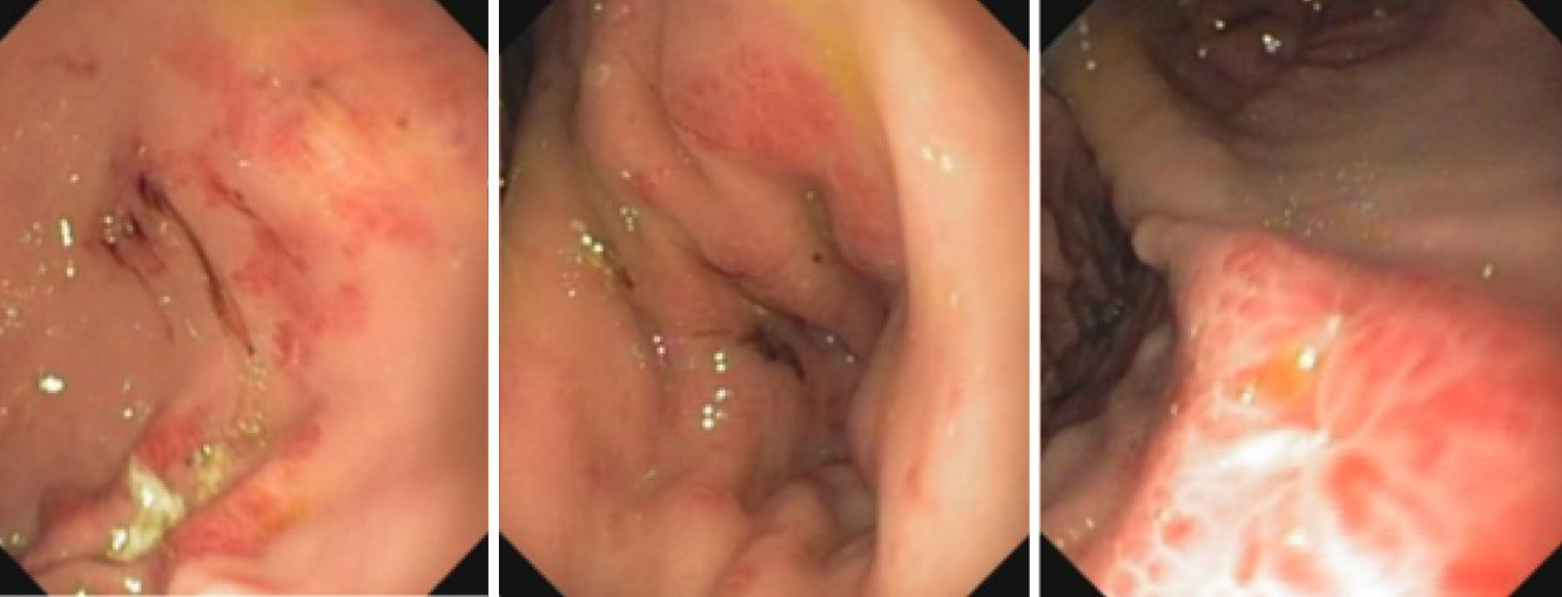
Eosinophilic gastroduodenitis, bulb mucosa

**Fig. 5:**
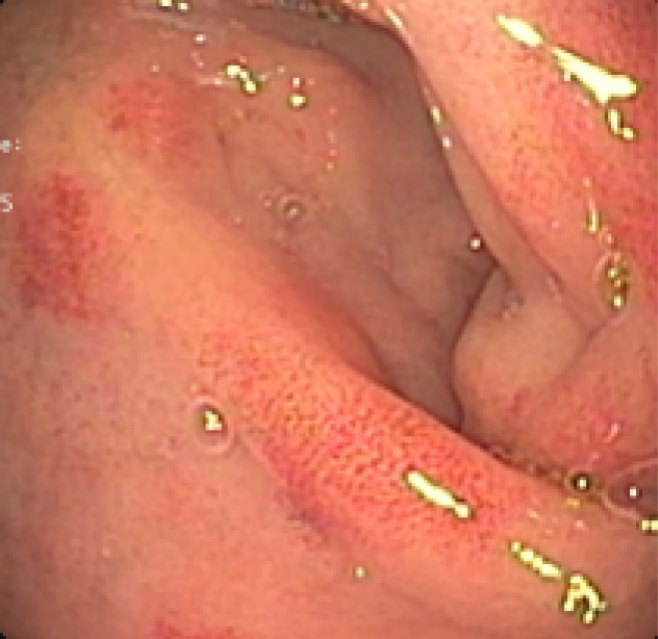
Eosinophilic colitis

Often, endoscopic aspects, either for stomach and for colon, can be normal or demonstrate non-specific aspects of chronic inflammation [70]. Mucosal erythema, friability, ulcerated mucosa, mucosal nodules or whitish specks can be also found [63] (Figs. 4, 5).

Diagnosis must be based on the finding of eosinophilic infiltration of mucosa. There is not a universally accepted threshold of eosinophils/HPF, in fact they are present in normal conditions in gastroenteric tract (only exclusion is esophagus). The most accepted definition is a count that exceeds 20 Eo/HPF [63, 70] in at least one sample. Besides eosinophils, a higher concentration of theirs mediator can be found in biopsies, in particularly metalloproteinases and eosinophilic cationic protease, IL-3, IL-5 and GMCSF [40, 63].

Since distribution of eosinophilic infiltration is not linear and can be present even in areas macroscopically normal, multiple biopsies are necessary [63].

## Diagnosis

Establishing a diagnosis of EGE is not easy: in fact, single laboratory tests or procedures that allow to identify this disease are lacking. Certainly, patients must present chronic or recurrent gastrointestinal symptoms, with documented eosinophilic infiltration of one or more gastrointestinal areas. Then, other causes of gastrointestinal eosinophilia and eosinophilic infiltration in other organs must be excluded.

If EGE is suspected, a blood sample must be collected. In fact, up to 80% of these patients presents peripheral eosinophilia. It appears to be more severe in patients affected by serosal subtype of EGE [71]. Anyway, it does not correlate with the activity of disease and it must not be used for follow up during or after therapy [72].

Moreover, before setting the diagnosis, it must be included [63]:

- Endoscopic and histological evaluations, that must present the characteristic mentioned above.
- Exclusion of other causes of gastrointestinal eosinophilia, such as hypereosinophilic syndrome, eosinophilic granulomatosis with polyangiitis (EGPA), celiac disease, inflammatory bowel disease, polyarteritis nodosa, other connective tissue disorders, infections, and drug hypersensitivity, through the use of careful collected history, laboratory testing and histological examination.

Once diagnosis of EGE is established, allergy evaluation must be carried out. In fact, up to 70% of patients with EGE can present a personal or family history of atopy, especially food allergy is very common [73]. Food allergies may be a trigger of clinical manifestations and/or pathogenesis of EGE. Atopy’s role is not yet well defined, also because EGE seems to be determined by both an IgE mediated mechanism and IgE-mediated- Th2 delayed hypersensitivity process. Anyway, allergen-specific in vitro or in vivo tests are indicated in these patients, because, if positive, elimination of causative foods is effective on symptoms [74].

## Therapy

Treatment of EGE includes dietetic and pharmacological approach. Surgery or other kind of therapy (i.e. paracentesis) may be needed in case of complications.

### Dietetic therapy

Following the benefits obtained in patients with EoE, a dietetic approach was tested also in EGE subjects. Nowadays, data about efficacy of dietetic treatment are based on case reports or on small series of patients. Chehade demonstrated the effectiveness of elemental diet in six pediatric patients [75], while SFED diet was tested in one child with benefit [76]. Data available about IgE-targeted diet are discordant [63].

### Pharmacological therapy

Corticosteroids therapy represents the main therapy for EGE and is effective in about 80% of patients [38]. There are not randomized trials for EGE therapy. In literature the most common first approach is systemic corticosteroid therapy with 20–40 mg/daily of prednisone for 2–6 weeks. In some cases this treatment was then continued with topic budesonide with benefit [77–79].

Other therapeutic approaches (mast cell stabilizers, omalizumab, mepolizumab, reslizumab, infliximab, leukotriene inhibitors, antihistaminic drugs, Th-2 inhibitors, such as suplatast and CRTH2) have been investigated, but their efficacy is not yet well defined, even because of the small number of patients involved.

## Abbreviations

BANCR: BRAF-activated non-coding RNA
CAPN14: calpain 14
DSG1: desmoglein 1
EoE: eosinophilic esophagitis
EGE: eosinophilic gastroenteritis
EGIDs: eosinophilic gastrointestinal diseases
EGPA: eosinophilic granulomatosis with polyangiitis
GERD: gastroesophageal reflux disease
GM-CSF: granulocyte-macrophage colony-stimulating factor
HPF: high power field
iNKT: invariant natural killer T
NGF: neurotropic tyrosine kinase
NTRK1: neurotropic tyrosine kinase receptor type 1
PPI: proton pump inhibitor
SFED: six food elimination diet
SNP: single nucleotide polymorphism
SPT: skin prick test
TGF- β: transforming growth factor-beta
TNF-α: tumor necrosis factor alpha
TSLP: thymic stromal lymphopoietin
